# Association between exposure to particulate matter during pregnancy and birthweight: a systematic review and a meta-analysis of birth cohort studies

**DOI:** 10.7555/JBR.31.20170038

**Published:** 2017-05-30

**Authors:** Yinwen Ji, Fei Song, Bo Xu, Yining Zhu, Chuncheng Lu, Yankai Xia

**Affiliations:** 1. State Key Laboratory of Reproductive Medicine, Institute of Toxicology, Nanjing Medical University, Nanjing, Jiangsu 211166, China; 2. Key Laboratory of Modern Toxicology of Ministry of Education, School of Public Health, Nanjing Medical University, Nanjing, Jiangsu 211166, China; 3. Department of Research and Education, The Children's Hospital, Zhejiang University School of Medicine, Hangzhou, Zhejiang 310003, China; 4. Department of Epidemiology and Biostatistics, School of Public Health, Tianjin Medical University, Tianjin 300070, China; 5. Department of Thoracic Surgery, The First School of Clinical Medicine, Nanjing Medical University, Nanjing, Jiangsu 210029, China.

## Abstract

Studies of the associations between maternal exposure to particulate matter (PM) and risk of adverse effects on fetal growth are inconsistent and inconclusive. This question can be well answered by carefully designed birth cohort studies; however, so far the evidence from such studies has not come to the same conclusion. We sought to evaluate the association between maternal exposures to PM and low birthweight (LBW) enrolling 14 studies from 11 centers, and to explore the influence of trimester and exposure assessment methods on between-center heterogeneity in this association. Data were derived from PubMed, Embase, Google Scholar, CNKI, and WanFang database, references from relevant articles, and results from published studies until March 2017. Using a random-effects meta-analysis, we combined the coefficient and odds ratios (OR) of individual studies conducted among 14 birth cohort studies. Random-effect meta-analysis results suggested that a 17% and 6% increase in risk of LBW was relevant to a 10 μg/m^3^ rise in PM_2.5_ and PM_10_ exposure concentrations at the 3rd trimester (pooled odds ratios (OR), 1.17 and 1.06; 95% confidence interval (CI), 0.94–1.46 and 0.97-1.15, respectively), but the null value was included in our 95% CI. Our results showed that exposure to PM_2.5_ and PM_10_ during pregnancy has a positive relevance to LBW based on birth cohort studies. However, neither reached formal statistical significance. Negative impacts on outcomes of birth is implied by maternal exposure to PM. Further mechanistic researches are needed to explain the connection between PM pollution and LBW.

## Introduction

Ambient particulate matter (PM) pollution contains PM_10_ (aerodynamic diameter <10 μm) and PM_2.5_ (aerodynamic diameter <2.5 μm), has been greatly associated with some negative health outcomes, including morbidity and mortality of cardiovascular^[1–2]^ or respiratory diseases^[3–4]^. In recent 20 years, the relationship between ambient PM and birth outcome has been the subject of much epidemiological research^[5–10]^.

Birthweight is a characteristic indicator of prenatal growth, and infants with low birthweight (LBW) is one of the adverse pregnancy outcomes. Infants with LBW are at a greater risk of mortality and morbidity than infants with normal birthweight, as well as having health issues in childhood and beyond. These effects include asthma, hypertension and compromised cognitive ability^[11–13]^. Accordingly, exploring the risk factors for LBW, in order to reduce the occurrence of LBW, is extremely important to public health. The connection between heavy air pollutants, exposure during pregnancy, and LBW in recent years are investigated by numerous studies. Laurent *et al*. found that LBW was positively and significantly associated with the zone but not total fine PM^[14]^. Similarly, a national study conducted in Canada discovered that there was a steady indication of a dose-response association for NO_2_ but no PM_2.5_ impact on LBW^[15]^. Also, daily PM_2.5_ with individual gestational ages of births in the contiguous United States was linked by another national study. This study indicated no overall significant positive connection between LBW and PM_2.5_ exposure during pregnancy^[16]^.

A number of researches explored significant relationships between PM exposure and LBW^[17–18]^. These inconsistent and controversial results suggest that quantitatively integration and interpretation of available evidence produce more accurate results for policy decisions and clinical need is necessary.

Results from several independent studies can be quantitatively integrated using meta-analysis, a most commonly used statistical method^[19]^. The relationship between PM exposure of pregnant women and neonatus birthweight has also been quantitatively analyzed by a number of meta-analyses^[6,20–21]^. However, all these meta-analyses observed notable heterogeneity among the studies included. Therefore, integrating various study results was necessary through a meta-analysis.

Here, we collected several studies that evaluated the effects of PM (PM_2.5_ and PM_10_) exposure during pregnancy on LBW, followed by employing a model of meta-analysis to estimate the effects of PM exposure on LBW during various pregnancy phases based upon birth cohort studies. The study site, sample, publish year, and exposure measurement methods were further evaluated for their potential influence on our meta-analysis.

Birth cohort studies provide the strongest evidence to comprehend the incidence and progression of diseases made possible by frequent follow-up data. None of the systematic reviews to date have paid special attention to the evidence from birth cohorts. Therefore, we present a systematic review and meta-analysis to estimate the effects of PM exposure during the different gestational periods with LBW.

## Materials and methods

### Search strategy

We perform and report the corresponding results in this meta-analysis based on the Preferred Reporting Items for Systematic Reviews and Meta-analyses (PRISMA) guidelines^[22]^.

All publications indexed in English-language databases including PubMed, Google Scholar, and Embase; as well as Chinese-language databases such as China National Knowledge Infrastructure (CNKI), WanFang databases before March 6, 2017, were systematically searched for studies which can be included into our meta-analysis. A combination of the following keywords were used for searching the relevant literatures: ("air pollution" OR "particulate matter" OR "fine particulate matter" OR "fine particles" OR "PM" OR "PM_10_" OR "PM_2.5_") AND ("cohort" OR "observational" OR "longitudinal" OR "follow-up") AND ("birth weight" OR "BW" OR "change in birth weight" OR "low birth weight" OR "LBW" OR "term low birth weight" OR "TLBW" OR "adverse birth outcomes" OR "adverse pregnancy outcomes"). Simultaneously, the references of relevant publications and meta-analysis were also investigated manually.

### Selection criteria

Eligible studies included were considered if they satisfied the following conditions: (1) using cohort study design (e.g., not descriptive study, case-control design and experimental design, randomized controlled trial, etc.); (2) LBW was defined as a live birth weighing less than 2500 g, including term LBW (TLBW) and preterm LBW (PLBW); (3) sample size, partial regression coefficient (β) for birthweight, usable risk estimates (e.g., odds ratio (OR), risk ratio (or relative risk, RR) or necessary data for calculation) for LBW, and its 95% confidence intervals (CI), or necessary information from which these results could be inferred; (4) other risk factors that could impact the outcomes of pregnancy had to be modified, including but not limited to maternal age and infant sex. If a birth season, maternal tobacco, or alcohol consumption during pregnancy and socioeconomic status were also modified; (5) only publications in English or Chinese were considered.

In the last step, we excluded the birth records directly from the database or national public health system, and sources of PM pollution from indoor were also ignored.

### Quality evaluation

Independently, JY and SF conducted a quality assessment of each study, included in our study, referring to the criteria derived from the Newcastle-Ottawa Scale (NOS)^[23]^. The NOS, which is used for assessing the quality of nonrandomized studies in meta-analyses, is available at: http://www.ohri.ca/programs/clinical_epidemiology/oxford.asp. All literature included in this study were cohort studies. The items of NOS score for cohort studies was divided into three domains: selection of cohort (four points), comparability of the cohort (two points), and assessment of outcome (three points). The quality of the study was considered high or moderate if the sum score was eight points or greater or between five and seven points, respectively. This ensured that each of the eligible articles was of high or moderate quality.

### Data extraction

Data was extracted from all eligible studies by two independent researchers (JY and SF) based on a standardized form. We resolved discrepancies by discussing with a third researcher (XB) extracting information consisting of the first author's name, year of publication, study location, study name, sample size, pollutant, exposure assessment, PM exposure windows (if a research implied connection between PM exposure during the whole pregnancy and/or trimester-specific periods and low birthweight, the assessments were obtained completely), outcome definition, covariates in the final model, and OR/RR/hazard ratio estimates with corresponding 95% CIs for all categories, continuous exposure of interest, or both, from each included research.

As gaseous pollutants were often different among studies, we obtained assessments from models of a single pollutant for results that covariates were fully modified. In this meta-analysis, we preferred the results that would depend on a larger number of pollutants.

For instance, we extracted the study of Michael Brauer *et al. *^[24–25]^ over those from^[26]^ for the results because the former study covered both PM_2.5_ and PM_10_ for the assessment method based on the monitoring network being more common across present studies. We preferentially chose this method to potentially reduce the heterogeneity among researches in this study.

### Meta-analysis and statistical analysis

Various studies have been reported with different increments (e.g., increased with an interquartile range) or compared to a reference category. To pool estimates from the studies enrolled in, all risk estimates (OR) of PM_10_ or PM_2.5,_ mass concentrations were converted to a uniform exposure of 10 μg/m^3^. Effect estimates were categorized by gestational period (whole pregnancy and trimester-specific). Weighting the inverse of the variance, we used a random-effects model to compute the pooled ORs and corresponding 95% CIs for each outcome of interest. Thereafter, the I^2^ statistic test was assessed to evaluate heterogeneity among estimates from primary studies (50% or less for low-, 51%–75% for moderate-, and 76% or more for high-heterogeneity), respectively. We also performed a series of sensitivity analyses by removing study singly to examine whether the results were strongly influenced by a specific study. Finally, publication bias might have existed and was detected with conducting funnel plot asymmetry, and then we evaluated funnel plot symmetry through Egger's regression.

All two-sided tests were of α = 0.05. Statistically significant findings were considered as those with a p-value<0.05. We performed all statistical analyses with Stata version 11.0.

## Results

### Search results and study characteristics

**Fig.1:**
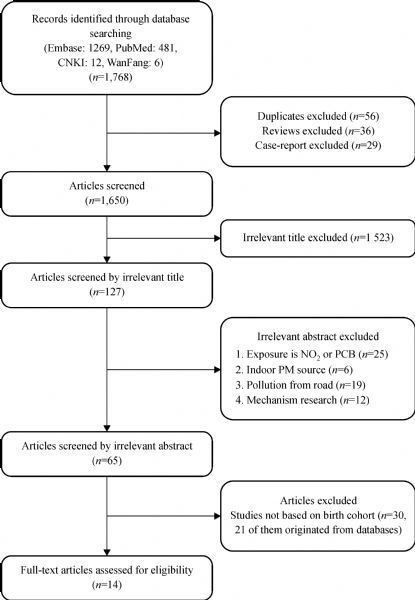
Flow chart of selection of studies

After a systematic search and review, we initially searched a total of 1,768 published English and Chinese literature; however, after excluding duplicates, reviews, and case-report articles, 1,650 kinds of literature remained. After glancing at these titles, 1,523 articles were further excluded as they were considered inappropriate regarding the interested endpoints or exposures. We reviewed the abstracts of the remaining 127 articles in detail. Sixty-two additional papers were removed since they focused on PCB/NO_2_ exposure to indoor pollution, pollutants from the road, or mechanism researches, leaving 65 articles for an in-depth review. A flow chart of the selection is shown in ***Fig. 1***. After carefully reviewing these articles, we distinguished fourteen for the final analysis from 2004 to 2016^[18,24,27–38]^, of which six studies assessed PM_2.5_ and LBW, twelve studies assessed PM_10_ and LBW, and three studies assessed both birthweight and LBW. ***Table 1*** (PM_2.5_-LBW) and ***Table 2*** (PM_10_-LBW) show the major features of the studies chosen for meta-analysis. Only six articles studied PM_2.5_ exposure for outcome LBW. Twelve studies used PM_10_ measures. Four studies provided evaluations for both pollutants. Three studies were from USA, and three studies from Canada, respectively. Others are from the UK, Netherlands, Tehran, Taiwan, Spain, Poland, Brazil, Korea and twelve European countries. Detailed information for all of the included studies can be seen in ***Tables 1*** and ***2***.

**Tab.1:** Characteristics of the studies included in the meta-analysis for PM_2.5_

Ref.	Year	First author	Study location	Study name	No. of birth	Pollutants measured	Exposure assessment	Exposure window		Adjustments	Outcomes	OR (95% CI)	
18	2013	Pedersen	12 European countries	European Study of Cohorts for Air Pollution Effects (ESCAPE)	74,178	PM_2.5_	LUR-model		WP	Gestational age, Sex, Parity, Maternal height, Weight before pregnancy, Maternal active smoking during second trimester, Maternal age, Maternal education, Season of conception	TLBW	1.18 (1.06, 1.33)	
24	2008	Brauer	Canada	Cohort	70,249	PM_2.5_	Monitor		WP	sex, ethnicity, parity, birth month and year, income, education	TLBW	1.03 (0.99, 1.07)	
30	2014	Ha	USA	retrospective cohort study	423,719	PM_2.5_	Hierarchical Bayesian Prediction Model			Maternal education, Ethnicity, Marital status, Maternal age, Infant gender, Prenatal care status, Alcohol, Smoking, Season of conception, Census group income, Urbanicity, presence or absence of maternal risk factor, Infection, PTD status, Co-morbidity	TLBW	W:1.015 (0.989, 1.041), T1:1.007 (0.981, 1.034), T2:1.034 (1.007, 1.061), T3:1.005 (0.980, 1.031)	
38	2014	Silva	Brazil	population-based retrospective cohort study	6,642	PM_2.5_	Model		WP,TS	Newborn sex, Type of delivery, Number of prenatal visits, Mother's education, Age group	TLBW	W: 1.33 (0.92, 1.90), T1:1.02 (0.74, 1.42), T2:1.51 (1.04, 2.17), T3: 1.50 (1.06, 2.15)	
32	2014	Dadvand	Spain	cohort	6,438	PM_2.5_	LUR-model		WP,TS	Neighborhood SES, Ethnicity, Education level, Marital status, Age, Smoking during pregnancy, Alcohol consumption during pregnancy, Admission BMI<20 kg/m2, Diabetes, Infection during pregnancy, Parity, Infant sex, Season and year of conception	TLBW	W:1.17 (0.98, 1.39), T1:1.07 (0.88, 1.29), T2:1.19 (0.97, 1.45), T3:1.24 (1.03, 1.49)	
33	2015	Poirier	Canada	retrospective cohort	13,400	PM_2.5_	LUR-model		WP	Maternal age, Parity, Smoking, Neighborhood income	TLBW	0.95 (0.89, 1.02)	
												

**Tab.2:** Characteristics of the studies included in the meta-analysis for PM_10_

Ref.	Year	First author	Study location	Study name/design	No. of birth	Pollutants measured	Exposure assessment	Exposure window	Adjustments	Outcomes	OR (95% CI)
27	2012	Araban	Tehran	Birth cohort	225	PM_10_	Monitor	WP,TS	Maternal age, Maternal education, Maternal job, Socioeconomic, Factor, Stress status, Number of prenatal, Care visits, Weight gain during pregnancy, Gestational	TLBW	W: 0.63 (0.40,1.14), T1: 0.63(0.40,1.14), T2:0.63 (0.52,1.84), T3:0.73(0.32,1.64)


28	2012	Hooven	Netherlands	Birth cohort	7,772	PM_10_	Model	WP	Maternal age, Educational level, Parity, Folic acid supplementation use	LBW	1.00 (0.95, 1.05)
18	2013	Pedersen	12 European countries	European Study of Cohorts for Air Pollution Effects (ESCAPE)	74,178	PM_10_	LUR-model	WP	Gestational age, Sex, Parity, Maternal height, Weight before pregnancy, Maternal active smoking during second trimester, Maternal age, Maternal education, Season of conception	TLBW	1.16 (1.00,1.35)
35	2006	Dugandzic	Canada	Birth cohort	74,284	PM_10_	Monitor	T1, T2,T3	Birth year, Gender of infant, Gestational age, Maternal age, Parity, Smoking during pregnancy, Weight change, Prior neonatal deaths, Prior stillbirth, Prior low birth weight, Neighborhood family income	TLBW	T1:1.03 (0.94, 1.14), T2:1.00 (0.90, 1.10), T3:0.94 (0.85, 1.05)
34	2004	Lin	Taiwan	Birth cohort	128,512	PM_10_	Monitor	WP,TS	Gestational week, Gender, Birth order, Season of birth, Maternal age, Educational attainment, Concentrations of various air pollutants	TLBW	W: 0.87 (0.71–1.05), T1: 0.97 (0.80–1.17), T2:1.00 (0.83–1.21), T3: 0.97 (0.81–1.17)
29	2005	Salam	USA	Children's Health Study(CHS)	3,901	PM_10_	Monitor	WP,TS	Maternal age, Months since last live birth, Parity, Maternal smoking during pregnancy, SES, Marital status at childbirth, Gestational diabetes, Child's The file type is not allowed. Please review the instructional text for allowable file types. sex, Race/ethnicity	LBW	W:1.3 (0.8, 2.2), T1:1.0 (0.7,1.5), T2:1.2 (0.8,1.7), T3:1.3 (0.9, 1.9)
24	2008	Brauer	Canada	Birth cohort	70,249	PM_10_	Monitor	WP	Sex, Ethnicity, Parity, Birth month and year, Income, Education	TLBW	1.01 (0.95,1.08)
31	2015	Dibben	Scotland	Scottish Longitudinal Study(SLS)	21,843	PM_10_	Model	WP	Social class, Parity, Individual estimated income, Ethnicity, Smoking, Mother's age, Mother's education, Season of birth	TLBW	1.07 (1.01,1.14)
36	2011	Xu	USA	Birth cohort	100,595	PM_10_	Monitor	TS	Maternal age, Maternal race, Maternal education, Smoking, Weight gain, Gender of infant, Gestation age, Parity, Previous LBW or Preterm birth, Level of prenatal care and birth season	TLBW	T1:1.13(1.02,1.25), T2: 1.10(1.00,1.22), T3:1.05(0.96,1.16)
37	2007	Kim	Korea	Birth cohort	1,514	PM_10_	Monitor	TS	Infant sex, Infant order, Maternal age and education level, Paternal education level, Season of birth, Alcohol drinking, Maternal body mass index (BMI) and maternal weight	TLBW	T1:1.1 (1.0, 1.2), T3:1.1 (1.0, 1.2)
32	2014	Dadvand	Barcelona	Birth cohort	6,438	PM_10_	LUR-model	WP,TS	Neighborhood SES, Ethnicity, Education level, Marital status, Age, Smoking during pregnancy, Alcohol consumption during pregnancy, Admission BMI<20 kg/m2, Diabetes, Infection during pregnancy, Parity, Infant sex, Season, Year of conception	TLBW	W:1.16 (0.98, 1.37), T1:1.00 (0.82, 1.22), T2:1.20 (0.96, 1.48), T3:1.26 (1.06, 1.51)
33	2015	Poirier	Canada	Birth cohort	13,400	PM_10_	LUR-model	WP	Maternal age, Parity, Smoking, Neighborhood income	TLBW	0.93 (0.88, 0.98)

### The quality of included studies

***Table 3*** presents the study-specific quality according to Newcastle-Ottawa quality scale.

**Tab.3:** Quality assessment using Newcastle-Ottawa quality assessment scale for the studies included in the meta-analysis

Source	Selection*	Comparability^†^	Outcome^‡^	Quality
Araban *et al*., 2012,Tehran(2)	★★★	★★	★★	Moderate
Kim *et al*., 2007, Korea(44)	★★★★	★★	★★	High
Hooven *et al*., 2012, Netherlands(4)	★★★	★★	★★	Moderate
Pedersen *et al*., 2013, 12 European countries(10)	★★★★	★★	★★	High
Dugandzic *et al*., 2006, Canada(17)	★★★★	★★	★	Moderate
Lin *et al*., 2004, Taiwan(18)	★★★★	★★	★★	High
Salam *et al*., 2015, USA(20)	★★★	★★	★	Moderate
Brauer *et al*., 2008, Canada(23)	★★★★	★★	★★	High
Ha *et al*., 2014, USA (25)	★★★	★★	★★	Moderate
Silva *et al*., 2014, Brazil (35)	★★★	★★	★★	Moderate
Dibben *et al*., 2015, UK(42)	★★★★	★★	★★	High
Xu *et al*., 2011, USA(43)	★★★	★★	★★★	High
Dadvand *et al*., 2014, Spain(53)	★★★★	★★	★	Moderate
Poirier *et al*., 2015, Canada (LING)	★★★★	★★	★★	High

*Stars awarded for representativeness of the birth cohort, selection of the normal birth cohort, PM exposure during pregnancy, the ascertainment of the diagnostic of the LBW. A maximum of four stars is to be awarded. ^†^Stars awarded for adjustment of related confounders. A maximum of two stars is to be awarded. ^‡^Stars awarded for assessment of LBW, length of follow-up, and adequacy of follow-up cohorts. A maximum of three stars is to be awarded.

### Pooled estimate of the effect of the PM mass concentrations on low birth weight

**Fig.2:**
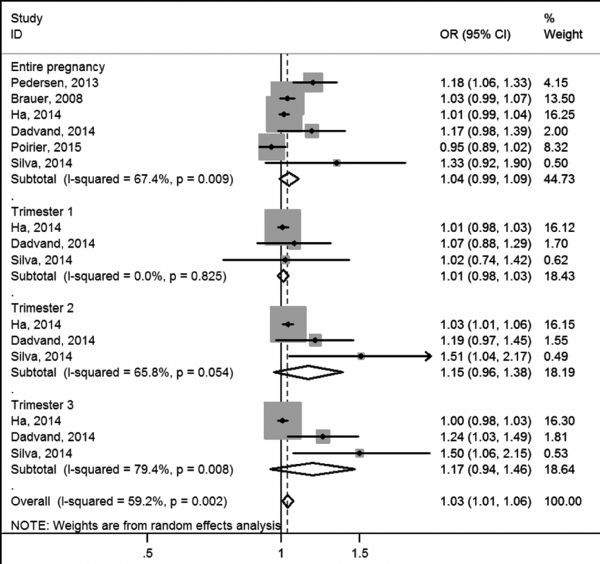
Meta-analysis of PM_2.5_ exposure and LBW

**Fig.3:**
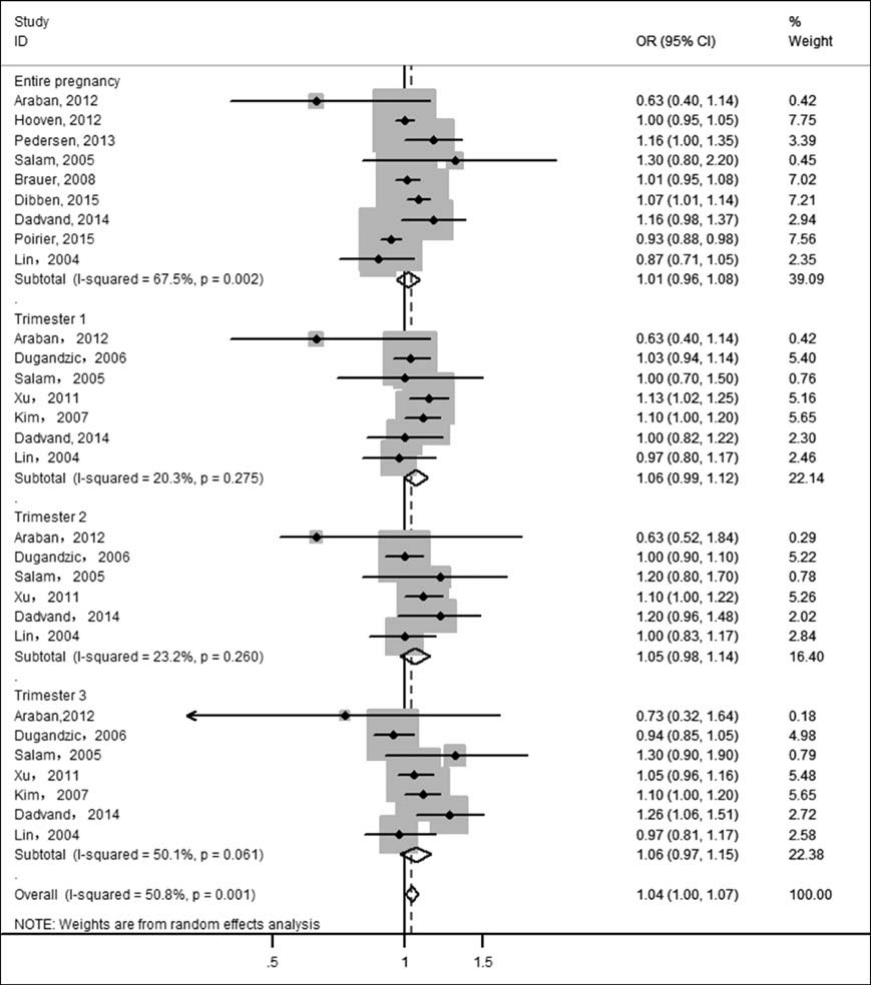
Meta-analysis of PM_10_ exposure and LBW

In ***Fig. 2*** and ***Fig. 3***, we estimated overall risks and the risk of LBW caused by PM_2.5_ was 1.03 (pooled OR, 95% CI: 1.01-1.06) and pooled OR of PM_10_ was 1.04 (95% CI: 1.00-1.07). Heterogeneity test indicated a moderate heterogeneity among six and twelve articles respectively, which was 59.2% for PM_2.5_-LBW ( *P* = 0.002) and 50.8% for PM_10_-LBW ( *P* = 0.001). Random effects model showed summarized ORs and 95% CIs: for PM_2.5_ during the entire pregnancy: 1.04 (0.99,1.09); 1st trimester: 1.01 (0.98,1.03); 2nd trimester: 1.15 (0.96, 1.38) and 3rd trimester: 1.17(0.94, 1.46); for PM_10_ during the entire pregnancy: 1.01 (0.96,1.08); 1st trimester: 1.06 (0.99,1.12); 2nd trimester: 1.05 (0.99, 1.44) and 3rd trimester: 1.06 (0.97, 1.15).

Another subgroup analysis was carried out by the study location (Europe and America; and other regions), study samples (≥1,000, and<1,000), published year (before 2010, and after 2010) and exposure measurement methods (monitor, and model) ( ***Table 4***). PM_2.5_ exposure with study sample below 10,000 (OR= 1.20, 95% CI: 1.101-1.299, I^2^ = 0.0%, *P* = 0.554), study sample above 10,000 (OR= 1.02, 95% CI: 1.00-1.042, I^2^ = 56.5%, *P* = 0.032), published year before 2010 (OR= 1.03, 95% CI: 0.991-1.071, I^2^ = 0.0%, *P* = 0.730), after 2010 (OR= 1.034, 95% CI: 1.007-1.061, I^2^ = 61.8%, *P* = 0.001); PM_10_ exposure with study sample below 10,000 (OR= 1.08, 95% CI: 1.00-1.15, I^2^ = 45.8%, *P* = 0.027), study sample above 10,000 (OR= 1.02, 95% CI: 0.98-1.06, I^2^ = 54.3%, *P* = 0.008), published year before 2010 (OR= 1.028, 95% CI: 0.99-1.067, I^2^ = 13.5%, *P* = 0.302), after 2010 (OR= 1.047, 95% CI: 0.988-1.11, I^2^ = 68.1%, *P*<0.001), study location at Europe and America (OR= 1.05, 95% CI: 1.01-1.09, I^2^ = 54.2%, *P* = 0.003), at Asia (OR= 0.98, 95% CI: 0.90-1.07, I^2^ = 48.6%, *P* = 0.041), exposure measurement methods with monitor (OR= 1.03, 95% CI: 0.99-1.08, I^2^ = 32.7%, *P* = 0.079), with model (OR= 1.05, 95% CI: 0.99-1.11, I^2^ = 70.3%, *P* = 0.001).

**Tab.4:** Subgroup analysis of the associations between maternal exposure to particulate matter (PM_2.5_ and PM_10_) and LBW

Pollutant	Sub-group	Division	I^2^	*P-*value	pooled OR (95% CI)
PM_10_	Period	Entire pregnancy	67.5%	0.002	1.01 (0.96, 1.08)
		Trimester 1	20.3%	0.275	1.06 (0.99, 1.12)
		Trimester 2	23.2%	0.260	1.05 (0.98, 1.14)
		Trimester 3	50.1%	0.061	1.06 (0.97, 1.15)
	Study area	Asia	48.6%	0.041	0.98 (0.90, 1.07)
		Europe and America	54.2%	0.003	1.05 (1.01, 1.09)
	Study Sample	≥10,000	54.3%	0.008	1.02 (0.98, 1.06)
		<10,000	45.8%	0.027	1.08 (1.00, 1.15)
	Published year	≤2010	13.5%	0.302	1.028 (0.99, 1.067)
		>2010	68.1%	<0.001	1.047 (0.988, 1.11)
	Assessment	monitor	32.7%	0.079	1.03 (0.99, 1.08)
		model	70.3%	0.001	1.05 (0.99, 1.11)
	Overall		50.8%	0.001	1.04 (1.00, 1.07)
PM_2.5_	Period	Entire pregnancy	67.4%	0.009	1.04 (0.99, 1.09)
		Trimester 1	0.0%	0.825	1.01 (0.98, 1.03)
		Trimester 2	68.8%	0.054	1.15 (0.96, 1.38)
		Trimester 3	79.4%	0.008	1.17 (0.94, 1.46)
	Study Sample	≥10,000	56.5%	0.032	1.02 (1.00, 1.042)
		<10,000	0.0%	0.554	1.20(1.101,1.299)
	Published year	≤2010	0.0%	0.730	1.03(0.991,1.071)
		>2010	61.8%	0.001	1.034(1.007,1.061)
	Overall		59.2%	0.002	1.03 (1.01, 1.06)

**Fig.4:**
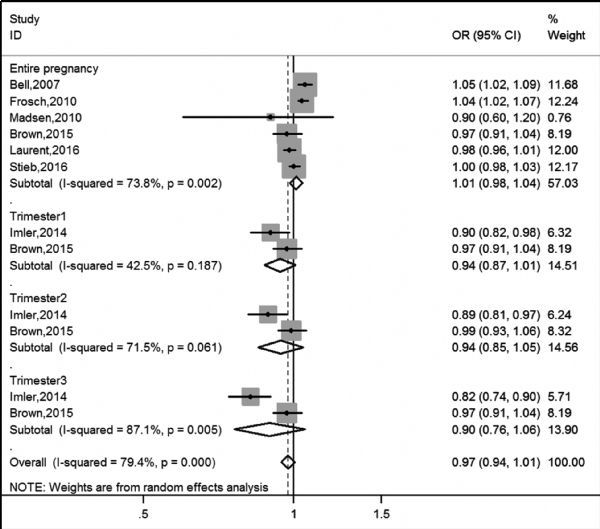
Meta-analysis of PM_2.5_ exposure and LBW based on birth data directly from national birth registry or hospital-birth records

**Fig.5:**
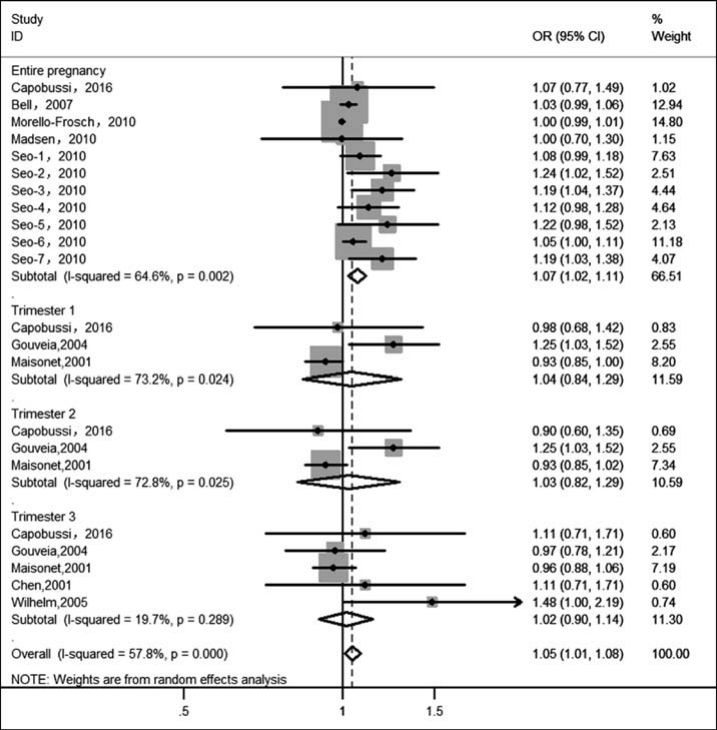
Meta-analysis of PM_10_ exposure and LBW based on birth data directly from national birth registry or hospital-birth records

On the other hand, we collected articles which used birth data directly from the national birth registry or hospital-birth records to explore the connection between PM exposure during pregnancy and LBW; the results were displayed in ***Fig. 4*** and ***Fig. 5***. The pooled the estimate of PM_10_ for the entire pregnancy (OR= 1.07, 95%:1.02, 1.11) was larger than other trimesters, although no statistical significance of the three estimates can be obtained. We also found that heterogeneity was the lowest for the 3rd trimester and the highest for the 1st trimester in ***Fig. 5***.

According to Egger's tests, except for the *P*-value ( *P* = 0.025) of PM_2.5_ exposure in the 3rd trimester, no significant publication bias for the two pollutants can be seen in ***Table 5***.

**Tab.5:** Publication bias during the entire pregnancy and trimesters were tested by Egger's test

Pollutant and LBW	Period	Egger's			
		t	*P*		
PM_10_	Entire pregnancy	0.31		0.764	
	Trimester 1	-2.63		0.050	
	Trimester 2	-0.79		0.475	
	Trimester 3	0.17		0.870	
PM_2.5_	Entire pregnancy	1.43		0.225	
	Trimester 1	1.18		0.447	
	Trimester 2	6.30		0.100	
	Trimester 3	25.3		0.025	

## Discussion

Fourteen eligible studies were identified and collected in our meta-analysis, and the associations between the PM mass concentrations and the risk of LBW were quantitatively assessed based on birth cohort studies. Results suggested that maternal exposures to PM during the entire pregnancy and trimesters had a slight positive trend to associate with LBW, but the results were not statistically significant, which consisted well with the findings of the previous meta-analyses. Sapkota *et al*. reported that slight but formally non-statistically significant increases in risk of LBW was associated with the entire pregnancy PM_2.5_ (summarized OR: 1.09; 95% CI: 0.90, 1.32) and PM_10_ exposure (summarized OR: 1.02; 95% CI: 0.99, 1.05)^[39]^. Although Stieb *et al*.^[6]^ reported that with a 10 μg/m^3^ increase for PM_2.5_ or a 20 μg/m^3^ increase for PM_10_, the pooled odds ratios concerning entire pregnancy exposure of PM_2.5_-LBW and PM_10_-LBW were 1.05 (0.99–1.12) and 1.10 (1.05–1.15).

To the best of our knowledge, this is the first meta-analysis study so far reporting on the association between PM pollution and LBW based on birth cohort. All of the studies enrolled in this meta-analysis were birth cohort records and most of these studies did not show an association of statistical significance between PM exposure and LBW. We supposed the reason for the difference lies in that Stieb included the articles based on a national birth registry or state center databases. To verify our supposition, we utilized 21 studies which were directly from databases of various centers. 

The in-depth evaluation of the evidence from birth cohorts is one of the main strengths of this review. If all articles were based on registered data, we could not get information comprehensively. A cohort study is an effective way to demonstrate the associations.

More or less, this meta-analysis has some limitations; although less heterogeneity in some subgroups, high or moderate heterogeneities appeared in many of the subgroup analyses. These findings illustrated that the heterogeneity may also be affected by other factors. The socioeconomic status were not investigated due to the limitation in quantity of relevant studies. Accordingly, further studies are warranted to examine the origins of heterogeneity as more meaningful studies are conducted in the future.
